# Leishmaniasis among Gold Miners, French Guiana

**DOI:** 10.3201/eid1207.051466

**Published:** 2006-07

**Authors:** Brice Rotureau, Michel Joubert, Emmanuel Clyti, Félix Djossou, Bernard Carme

**Affiliations:** * Université des Antilles et de la Guyane, Cayenne, French Guiana;; † Centre Hospitalier Andrée Rosemon, Cayenne, French Guiana

**Keywords:** Leishmania, cutaneous leishmaniasis, outbreak, epidemiology, French Guiana

**To the Editor:** In 2004, the Cayenne General Hospital and public health centers recorded 348 new cases of cutaneous leishmaniasis (CL) in French Guiana (*1*). A case of CL was considered confirmed if cutaneous lesions were present for >2 weeks; the patient had a compatible epidemiologic history; and microscopic examination of dermal scrapings, parasite cultivation, or both showed positive results for *Leishmania*. According to the population estimate given by the French National Institute for Statistics and Economical Studies (INSEE, Cayenne), the incidence of CL in 2004 was 0.2%–0.4% and has been relatively stable since 1979 (*2**,**3*). However, when the annual number of cases per village were examined, new CL cases were heterogeneously distributed. Saint Elie, a gold-mining village in the inland neotropical forest, had an apparent incidence rate of 25.9% in 2004 and 28.9% in 2005 (Figure); risk for infection in this village was, on average, 65× higher than anywhere else in French Guiana. We tested samples from 12 random CL patients with a *Leishmania*-specific polymerase chain reaction–restriction fragment length polymorphism test that targeted the internal transcribed spacer 1 of ribosomal RNA genes with primers SSU-12103-D (5´-GGGAATATCCTCAGCACGT-3´) and 5.8S-13333-R (5´-CGACACTGAGAATATGGCATG-3´) (*4*). All these patients were infected with *Leishmania guyanensis*.

**Figure:**
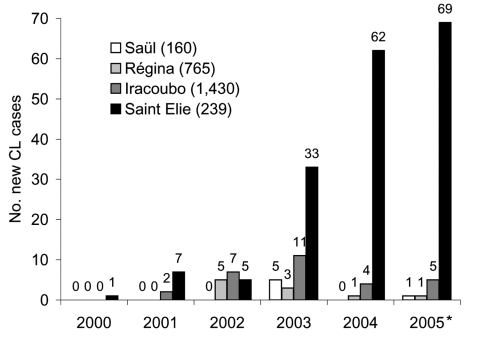
Number of new cutaneous leishmaniasis (CL) cases registered in health centers of 4 villages of French Guiana (Iracoubo, Régina, Saül, and Saint Elie) from 2000 to 2005. For each village, the 1999 population estimate (French National Institute for Statistics and Economical Studies, Cayenne) is given in parentheses. *Cases Jan–Aug 2005.

Isolated in dense rainforest (no road or airport) and with 239 inhabitants (INSEE, Cayenne), Saint Elie is situated on a gold seam; miners illegally create trails from the village to deposits in a 10-km circumference in the dense forest around the village. Compared to other French Guianan villages, such as Saül and Régina, which are similarly isolated in the rainforest and have 160 and 765 inhabitants (INSEE, Cayenne), respectively, and Iracoubo, the village closest to Saint Elie with 1,430 inhabitants (INSEE, Cayenne), substantially more new CL cases have been observed in Saint Elie since 2003. Since 2000, medical rounds have been undertaken every 15 days in the villages of Saint Elie and Saül, whereas people from Régina and Iracoubo have doctors at their disposal every day.

Official records indicate that the population of Saint Elie has doubled in the past 10 years, reaching 239 inhabitants in 1999 (INSEE, Cayenne). However, 860 new medical files have been registered in the Saint Elie Health Centre since 2000. This finding could be explained by the high number of illegal workers in this area. Patient interviews showed that most of these workers (≈90%) originated from the poorest northern Brazilian states (Pará, Amapá, Roraima, and especially Maranhão). Thus, the incidence rate of 25.9%, calculated on the basis of 239 inhabitants, was likely overestimated. Taking into account a substantial turnover in migrant populations, the denominator could be 500–1,000 inhabitants, and the incidence rate would be 6.2%–12.4%. All patients worked in the small-scale gold mines surrounding Saint Elie, and CL cases were recorded without seasonal fluctuations. Imported cases are possible, but reports are likely to be anecdotal because clinical observations, estimated dates of infection, and duration of patient stay in Saint Elie were congruent and because all genotyped strains were Guianan *L. guyanensis* (*1*).

Several infection risk factors exist simultaneously in this situation. In a CL-endemic area, immigrant populations, who are mostly nonimmune, exert pressure on the environment (deforestation) that directly increases their risk for exposure to infected vectors, in the absence of prophylactic measures. The initial short-term effect of deforestation is the mobilization of aggressive adult sandflies, which have been disturbed while resting. However, the ability of zoophilic vectors to adapt to peridomestic environments has also already greatly influenced the distribution of leishmaniases in South America (*5**–**7*).

Considering the uncertainty of the population estimate, turnover, and immunity status, we assume that incidence rates should be considered cautiously. Nevertheless, we found that gold mining in forested areas constitutes a risk factor for CL, at least in French Guiana and probably in all Amazonian rainforests. This risk could be a public health concern. Larger studies in other gold-mining areas are required to quantify the incidence of CL among workers to effectively focus prophylactic and preventive campaigns.
